# Retinal Vessel Responses to Flicker Stimulation Are Impaired in Ca_**v**_2.3-Deficient Mice—An *in-vivo* Evaluation Using Retinal Vessel Analysis (RVA)

**DOI:** 10.3389/fneur.2021.659890

**Published:** 2021-04-13

**Authors:** Felix Neumaier, Konstantin Kotliar, Roel Hubert Louis Haeren, Yasin Temel, Jan Niklas Lüke, Osama Seyam, Ute Lindauer, Hans Clusmann, Jürgen Hescheler, Gerrit Alexander Schubert, Toni Schneider, Walid Albanna

**Affiliations:** ^1^Department of Neurosurgery, RWTH Aachen University, Aachen, Germany; ^2^Forschungszentrum Jülich GmbH, Institute of Neuroscience and Medicine, Nuclear Chemistry (INM-5), Jülich, Germany; ^3^Institute of Radiochemistry and Experimental Molecular Imaging, Faculty of Medicine and University Hospital Cologne, University of Cologne, Cologne, Germany; ^4^Department of Medical Engineering and Technomathematics, FH Aachen University of Applied Sciences, Aachen, Germany; ^5^Department of Neurosurgery, Maastricht University Medical Center, Maastricht, Netherlands; ^6^Institute for Neurophysiology, University of Cologne, Cologne, Germany; ^7^Tranlational Neurosurgery and Neurobiology, RWTH Aachen University, Aachen, Germany

**Keywords:** *in vivo* retinal vessel analysis, neurovascular coupling, voltage- gated Ca^2+^ channels, dynamic retinal vessel analysis, Ca_v_2.3-deficient mice

## Abstract

**Objective:** Metabolic demand increases with neuronal activity and adequate energy supply is ensured by neurovascular coupling (NVC). Impairments of NVC have been reported in the context of several diseases and may correlate with disease severity and outcome. Voltage-gated Ca^2+^-channels (VGCCs) are involved in the regulation of vasomotor tone. In the present study, we compared arterial and venous responses to flicker stimulation in Ca_v_2.3-competent (Ca_v_2.3_[+/+]_) and -deficient (Ca_v_2.3_[−/−]_) mice using retinal vessel analysis.

**Methods:** The mice were anesthetized and the pupil of one eye was dilated by application of a mydriaticum. An adapted prototype of retinal vessel analyzer was used to perform dynamic retinal vessel analysis. Arterial and venous responses were quantified in terms of the area under the curve (AUC_art_/AUC_ven_) during flicker application, mean maximum dilation (mMD_art_/mMD_ven_) and time to maximum dilation (tMD_art_/tMD_ven_) during the flicker, dilation at flicker cessation (DFC_art_/DFC_ven_), mean maximum constriction (mMC_art_/mMC_ven_), time to maximum constriction (tMC_art_/tMC_ven_) after the flicker and reactive magnitude (RM_art_/RM_ven_).

**Results:** A total of 33 retinal scans were conducted in 22 Ca_v_2.3_[+/+]_ and 11 Ca_v_2.3_[−/−]_ mice. Ca_v_2.3_[−/−]_ mice were characterized by attenuated and partially reversed arterial and venous responses, as reflected in significantly lower AUC_art_ (*p* = 0.031) and AUC_ven_ (*p* = 0.047), a trend toward reduced DFC_art_ (*p* = 0.100), DFC_ven_ (*p* = 0.100), mMD_ven_ (*p* = 0.075), and RM_art_ (*p* = 0.090) and a trend toward increased tMD_art_ (*p* = 0.096).

**Conclusion:** To our knowledge, this is the first study using a novel, non-invasive analysis technique to document impairment of retinal vessel responses in VGCC-deficient mice. We propose that Ca_v_2.3 channels could be involved in NVC and may contribute to the impairment of vasomotor responses under pathophysiological conditions.

## Introduction

As brain tissue lacks significant energy reserves, proper global cerebral blood flow is critical for a constant supply of metabolic substrates, which is achieved through cerebral autoregulation, metabolic feedback mechanisms, and input from the autonomic nervous system (1). Much less is known about the exact mechanisms underlying neurovascular coupling (NVC), which mediates continuous adjustment of local cerebral blood flow to dynamic and regionally heterogeneous changes in neuronal activity and thus metabolic demand. NVC is accomplished by the so-called neurovascular unit (NVU), a subsumption of cell types with intimate anatomical and chemical relationship that comprises neuronal, glial, endothelial and vascular cells (2–5). Although still poorly understood, NVU function is increasingly recognized to be a complex multidimensional process that involves mediators released from the different cell types, which engage parallel signaling pathways across the entire cerebrovascular network (6, 7). In addition, there is strong evidence that dysfunctional signaling within the NVU (8) and in some cases even inversion of NVC (9) are involved in delayed cerebral ischemia and secondary brain damage after ischemic or hemorrhagic brain injury [reviewed in Guo and Lo (10)]. Voltage-gated Ca^2+^-channels (VGCCs) are critical for Ca^2+^ influx into all types of cells in the NVU and almost certainly involved in NVC under physiological and pathophysiological conditions (11, 12). L-type VGCCs have traditionally been regarded as the main pathway for Ca^2+^ entry into vascular smooth muscle cells (11) and they remain the only approved target for prevention of cerebral vasospasm after subarachnoid hemorrhage by the Ca^2+^ channel antagonist nimodipine (13). However, while L-type channels predominate in large caliber proximal vessels, there is growing evidence for a role of non-L-type VGCCs in smaller diameter resistance vessels (11, 14, 15). Their exact identity and functional relevance remains controversial, which may reflect the existence of significant heterogeneity among different vascular beds, pathophysiological changes in the functional expression of different channels and/or the expression of these channels in other cell types of the NVU (11, 12). In addition, interpretation of experimental results on NVU function is complicated by the fact that many results from *in vitro* studies appear to be inconsistent (7), highlighting the importance of non-invasive *in vivo* approaches for valid studies on the mechanisms underlying NVC. One such approach is to analyze vascular responses in the retina, which is an embryological part of the central nervous system that can be assessed non-invasively, so that it provides a unique “window to the brain.” Retinal vessel analysis (RVA) has been used in a number of clinical studies to assess microvascular responsiveness and to show that NVC is altered after ischemic or hemorrhagic stroke (16–18). In a previous, proof-of-principle study, we showed that RVA is also a feasible method for non-invasive assessment of vessel responses in the murine retina (19). In the present work, we used the same adapted prototype of a non-contact retinal vessel analyzer to investigate how genetic ablation of Ca_v_2.3 voltage-gated calcium channels affects arterial and venous retinal responses in mice. These channels have previously been implicated in delayed cerebral vasospasm and impaired NVC after subarachnoid hemorrhage (20), but their exact role for microvascular function remains to be elucidated. Our results suggest that Ca_v_2.3 channel dysfunction could be associated with altered NVC in the murine retina and demonstrate how RVA can be used for non-invasive *in vivo* studies on NVC in small animal models.

## Materials and Methods

### Animals

A total of 22 Ca_v_2.3-competent (Ca_v_2.3_[+/+]_) and 11 Ca_v_2.3-deficient (Ca_v_2.3_[−/−]_) male mice, aged 12–15 weeks, were used in the present study. Mice were housed in Makrolon type II cages at a constant temperature (20–22°C) with light on from 7 a.m. to 7 p.m. (light intensity at the surface of the animal cages was 5–10 lux) and *ad libitum* access to food and water. Ca_v_2.3_[−/−]_ mice were generated through deletion of exon 2 by Cre-mediated recombination (21). They are available from Mutant Mouse Resource and Research Centers (MMRRC) with the strain name B6J.129P2(Cg)-Cacna1etm1.1Tsch/Mmjax. Parallel breeding of parental inbred mouse lines of Ca_v_2.3_[−/−]_ and Ca_v_2.3_[+/+]_ mice ensured that they had the same mixed genetic background (C57Bl/6 × 129SvJ). The institutional and governmental committees on animal care [Landesamt für Natur, Umwelt und Verbraucherschutz [LANUV] Nordrhein—Westfalen, Recklinghausen, Germany; 84-02.04.2016.A4555] approved all experiments described in the text, which were conducted in accordance with accepted standards of humane animal care, as reported in the UFAW handbook on the care and management of laboratory animals.

### RCrodent Vessels Analysis

**Figure 1:**
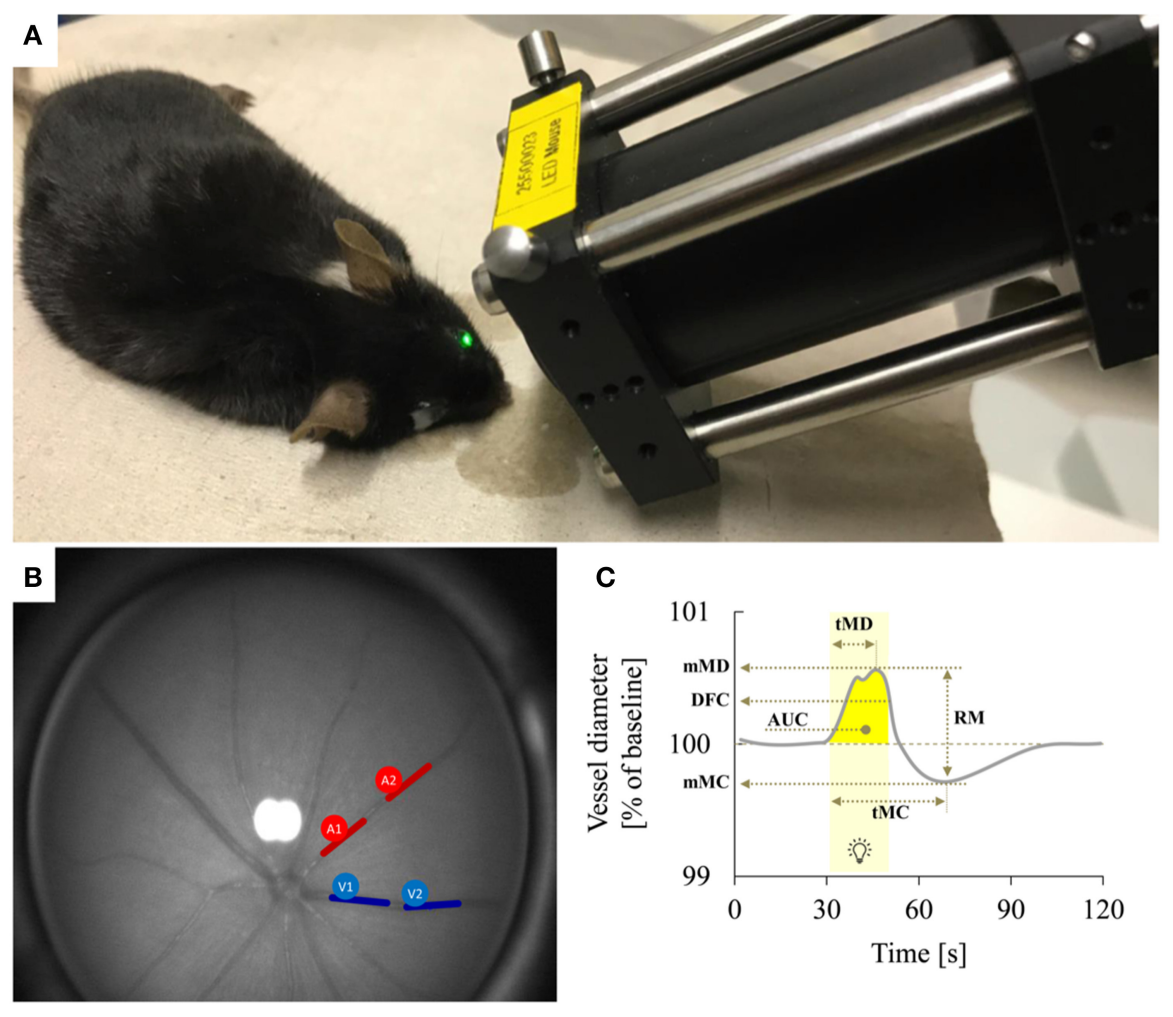
Dynamic retinal vessel analysis. **(A)** Experimental setup showing the murine retinal vessel analyzer (RCrodent, IMEDOS Systems UG, Jena, Germany) in operation. **(B)** Murine retina as assessed with the retinal vessel analyzer. Proximal and distal arterial (A1 and A2) and venous (V1 and V2) segments are marked in red and blue, respectively, as described in detail in text. **(C)** Idealized representation of a retinal vessel response to flicker stimulation with the different parameters determined in the present study. The stimulation period is indicated by light yellow shading. Parameters used for quantification of the response comprised mean maximum dilation during the flicker (mMD), time to maximum dilation (tMD) relative to flicker initiation, dilation at flicker cessation (DFC), mean maximum constriction after the flicker (mMC), time to maximum constriction (tMC) relative to flicker initiation, the area under the curve (AUC) during the flicker (indicated by dark yellow shading) and the reactive magnitude (RM), determined as the difference between mean maximum dilation during the flicker and mean maximum constriction after the flicker. For additional information on these parameters see Table 1.

The device used for vessel analysis was an adapted prototype of the Dynamic Vessel Analyzer (DVA) (RCrodent, IMEDOS Systems UG, Jena, Germany) previously used for other preclinical and clinical applications (22), which enables murine retinal vessel analysis as a function of time by applying flicker light impulses at defined frequencies like also reported in rats (23) (Figures 1A,B). Prior to the experiments, mice were dark-adapted overnight (12 h), anesthetized by intraperitoneal injection of ketamine (66.7 mg/kg body weight; Ketanest, Parke-Davis/Pfizer, Berlin, Germany) and xylazine (6.7 mg/kg body weight, Rompun 2% Bayer Vital, Leverkusen, Germany) and positioned on a heating plate to maintain a constant body temperature of 37°C. The left eye was then dilated by application of a mydriatic agent (Tropicamide, Mydriaticum Stulln UD, Pharma Stulln GmbH, Stulln, Germany) and equipped with a dedicated polymethylmethacrylate mouse contact lens (Back Optic Zone Radius 1.7 mm, diameter 3.2 mm, zero dioptric from Cantor & Nissel, Brackley, UK) to protect the cornea. For the measurements, we used a light intensity of 30 lux and the standard 350 s measurement protocol by IMEDOS Systems for human studies (24). After baseline assessment for 50 s, measurements were performed by application of three consecutive cycles of monochromatic rectangular flicker stimulation (530 nm, 12.5 Hz, 20 s). To assess retinal vessel diameters, we used the standard protocol and application mode of the DVA, where the fundus is illuminated during the whole measurement and the illumination light is diminished during the flicker stimulation with an application frequency of 12.5 Hz (19). This mode differs from alternative paradigms of visual stimulation in murine studies, where the application of light or other visual stimuli is preceded and followed by a measurement with no illumination (25).

Retinal arteries and veins were selected arbitrarily for RVA assessment in the murine fundus. In order to improve signal-to-noise ratio of the assessment, each vessel type of a mouse was attempted to be measured in two locations: proximal and distal to the optic nerve head (ONH) (proximal: >0.3 and <2.5 ONH plexus diameters from the ONH plexus rim, distal: > 2.5 and <5 ONH plexus diameters) as illustrated in Figure 1B. The individual retinal vessel parameters (Table 1) were then determined by taking the mean values from these proximal and distal measurements. This approach succeeded in 6/11 venous and 3/11 arterial vessels of Ca_v_2.3_[−/−]_ mice and in 18/22 venous and 6/22 arterial vessels of Ca_v_2.3_[+/+]_ mice. In the remaining cases, only one segment of each vessel type located in the area between 0.3 and 5 ONH plexus diameters from the rim could be assessed per animal.

**Table 1:** Definition of dynamic retinal vessel analysis parameters.

**Parameter**	**Unit**	**Meaning**
Arterial/Venous area under the curve during the flicker (AUC_art_/AUC_ven_)	%*s	Area under the response curve during the 20 s flicker stimulation
Mean maximal arterial/venous dilation in response to the flicker (mMD_art_/mMD_ven_)	% of baseline	Absolute maximum of the response to flicker stimulation
Time to maximal arterial/venous dilation during the flicker (tMD_art_/tMD_ven_)	s	Time between flicker initiation and the absolute maximum response during the flicker
Arterial/venous dilation at flicker cessation (DFC_art_/DFC_ven_)	% of baseline	Value of the response at the end of flicker stimulation
Mean maximal arterial/venous constriction after flicker cessation (mMC_art_/mMC_ven_)	% of baseline	Absolute minimum of the response curve after flicker cessation
Time to maximal arterial/venous constriction after flicker cessation (tMC_art_/tMC_ven_)	s	Time between flicker initiation and the absolute minimum response after flicker cessation
Arterial/venous reactive magnitude (RM_art_/RM_ven_)	% of baseline	Difference between mean maximal dilation during the flicker and mean maximal constriction after flicker cessation

In addition to the automated analysis performed by the commercial DVA software, further parameters of dynamic vascular response were derived and analyzed as described previously (26) using a template-set with macros (Microsoft Office Excel 2016, Californian, USA) to filter, process, and analyze numerical data from the original DVA-assessment (26, 27). Briefly, absolute vessel diameters within the retina were gauged by arbitrary measuring units (MU), where 1 MU corresponds to roughly 1 μm in the mouse eye. The conversion from the imaging system was adjusted to the size of the charge-coupled device (CCD) matrix and was calculated based on the size of a theoretical standard eye. For comparison of flicker responses between different animals, relative changes in vessel diameters were calculated in % of the individual baselines. To improve the signal-to-noise ratio for manual analysis, the three response curves obtained for each animal (30 s of baseline before flicker application, 20 s during flicker application and 80 s after flicker application) were averaged and smoothed using a running median with a time-window of 4 s and the corresponding back shift. The resulting curves were used to determine the parameters indicated in Figure 1C and Table 1.

Average time courses of vessel diameter changes in a given group were calculated according to a technique described previously (26, 28), where each time point of the average curve for the group is the median of all individual (relative) vessel diameters at this time point. The quality of DVA recordings was assessed semi-objectively using the cumulative scoring method described previously (27). The score ranged from 0 (“inadequate”) to 5 (“excellent quality”) and only DVA recordings with score values ≥ 2.0 were included in the analysis.

### Statistical Analysis

Unless noted otherwise, all data are shown as median [1st quartile−3rd quartile]. Statistical comparisons with the Mann-Whitney-U-test were carried out in an explorative manner without correction for multiple comparisons in order to show tendencies and to identify potential differences for further investigation. Statistical significance was set at *p* < 0.05 and statistical results with *p* ≤ 0.1 were considered as trend. All analyses and data presentation were performed with Excel (Microsoft Office Excel 2016, Californian, USA), SPSS v. 21 (IBM Chicago, Illinois, USA), and GraphPad Software (GraphPad Prism, Inc., La Jolla, USA). Boxplots show median values, 1st quartile and 3rd quartile (box), minimum and maximum values (whiskers), and individual data points (dots). Outliers were identified based on the definition by Turkey, where values below [1st quartile−1.5 ^*^ interquartile range (IQR)] or above [3rd quartile + 1.5 ^*^ IQR] are considered suspected outliers and values below [1st quartile−3 ^*^ IQR] or above [3rd quartile + 3 ^*^ IQR] are considered outliers.

## Results

The median body weight was 28.0 g (27.3 to 30.0 g) for Ca_v_2.3_[+/+]_ and 25.0 g (25.0 to 25.5 g) for Ca_v_2.3_[−/−]_ mice (*p* = 0.001).

**Figure 2:**
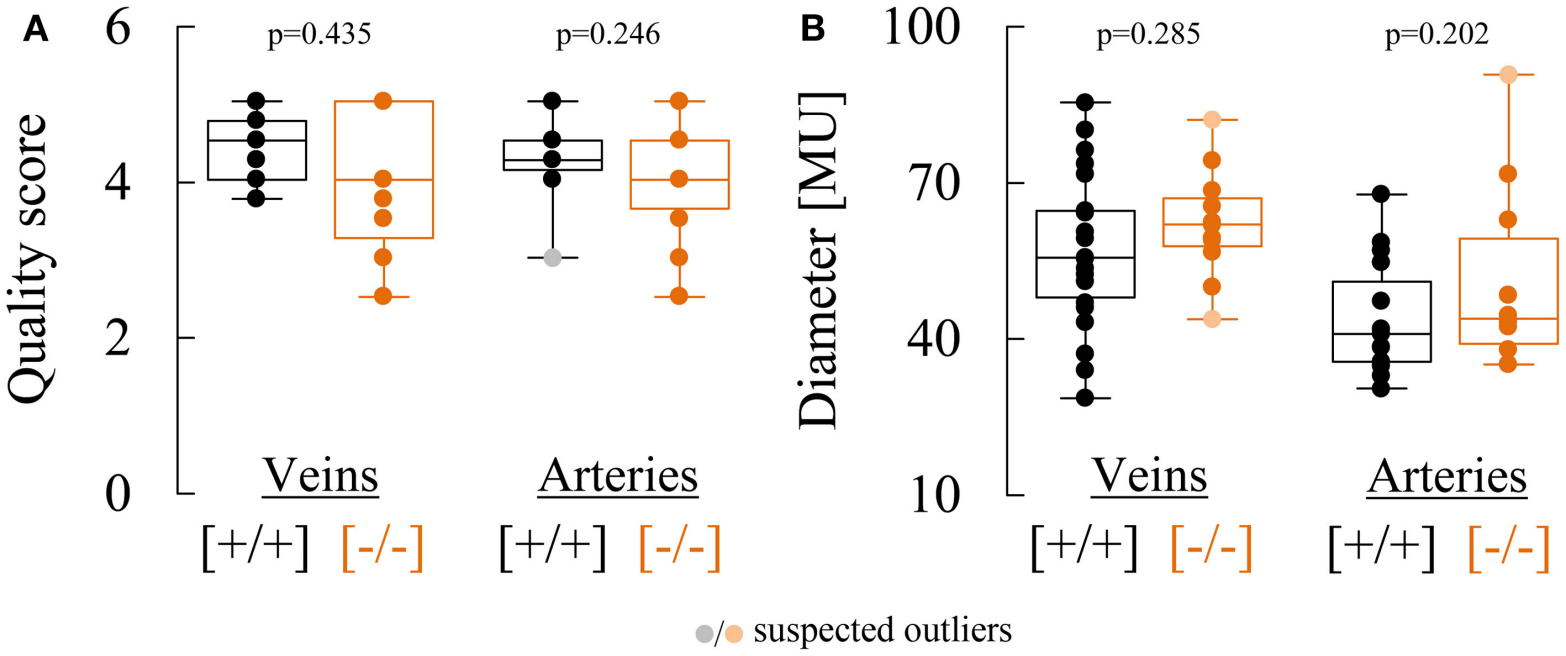
Comparison of retinal vessel diameters and data quality in Ca_v_2.3_[+/+]_ and Ca_v_2.3_[−/−]_ mice. **(A)** Comparison of semi-objective cumulative quality scores for venous and arterial responses recorded in Ca_v_2.3_[+/+]_ (black) or Ca_v_2.3_[−/−]_ (orange) mice. **(B)** Comparison of venous (left) and arterial (right) diameters determined in Ca_v_2.3_[+/+]_ (black) or Ca_v_2.3_[−/−]_ (orange) mice and expressed in arbitrary measuring units (MU). One MU corresponds to roughly 1 μm.

The response characteristics and values of the retinal vessel analysis parameters in the two genotypes are summarized in Tables 2, 3. The quality of the data was similar in both groups and sufficiently high for a comparative analysis (Figure 2A). There was no significant difference in median arterial [Ca_v_2.3_[+/+]_: 40.9 MU vs. Ca_v_2.3_[−/−]_: 43.9 MU, *p* = 0.202] or venous [Ca_v_2.3_[+/+]_: 55.5 MU vs. Ca_v_2.3_[−/−]_: 61.9 MU, *p* = 0.285] diameter between the two genotypes, although the values tended to be slightly higher in Ca_v_2.3_[−/−]_ mice (Figure 2B). As a measure for the retinal vascular density in both genotypes, we also calculated the overall number of vessels leaving the ONH in the murine fundus, which amounted to 10.0 (9.5 to 11.0) in Ca_v_2.3_[+/+]_ and 10.0 (10.0 to 10.0) in Ca_v_2.3_[−/−]_ mice (*p* = 0.486). Likewise, no difference was detected in the proportion of cases where one or more vessels branched in the near periphery to the ONH, which amounted to 86% in Ca_v_2.3_[+/+]_ compared to 82% in Ca_v_2.3_[−/−]_ mice (*p* = 0.630).

**Table 2:** Response characteristics of retinal veins and arteries in Ca_v_2.3_[+/+]_ and Ca_v_2.3_[−/−]_ mice.

**Response to flicker stimulation**	**Ca_**v**_2.3_**[+/+]**_ n (%)**	**Ca_**v**_2.3_**[−/−]**_ n (%)**	***p-*value**
**Veins**			**0.028**
No response	1 (5%)	3 (27%)	
Vasoconstriction	3 (14%)	4 (36%)	
Vasodilation	18 (82%)	4 (36%)	
All	22 (100%)	11 (100%)	
**Arteries**			**0.008**
No response	5 (23%)	0 (0%)	
Vasoconstriction	3 (14%)	7 (64%)	
Vasodilation	14 (64%)	4 (36%)	
All	22 (100%)	11 (100%)	

*Bold values indicate p ≤ 0.1*.

**Table 3:** Dynamic retinal vessel analysis parameters in Ca_v_2.3_[+/+]_ and Ca_v_2.3_[−/−]_ mice.

	**Ca_**v**_2.3[+/+] median (1.q−3.q)**	**Ca_**v**_2.3[–/–] median (1.q−3.q)**	***p-*value**
**General parameters:**	***n=*** **22**	***n=*** **11**	
Age, [months]	4.7 (4.0 to 5.7)	4.9 (3.5 to 6.0)	0.592
Weight, [g]	28.0 (27.3 to 30.0)	25.0 (25.0 to 25.5)	**0.001**
**Arterial parameters:**	***n=*** **15**	***n=*** **10**	
Data quality, [subjective score 1.0 to 5.0]	4.5 (4.0 to 5.0)	4.5 (3.8 to 5.0)	0.246
Arterial diameter, [MU]	40.9 (35.5 to 50.9)	43.9 (39.0 to 59.2)	0.202
Mean maximal arterial dilation (mMD_art_), [% baseline]	1.3 (0.6 to 2.2)	0.5 (0.2 to 1.2)	0.127
Time to maximal arterial dilation (tMD_art_), [s]	10.0 (8.0 to 14.3)	15.0 (10.8 to 25.3)	**0.096**
Arterial dilation at flicker cessation (DFC_art_), [% baseline]	0.3 (−0.4 to 0.5)	−0.1 (−0.7 to 0.1)	**0.100**
Arterial reactive magnitude (RM_art_), [% baseline]	3.1 (2.4 to 3.7)	1.8 (1.4 to 2.9)	**0.090**
Arterial AUC during the flicker (AUC_art_), [%*s]	2.3 (−1.7 to 10.0)	−4.5 (−7.3 to 1.9)	**0.031**
Mean maximal arterial constriction (mMC_art_), [% baseline]	−0.3 (−1.0 to 0.6)	0.4 (−1.1 to 1.0)	0.396
Time to maximal arterial constriction (tMC_art_), [s]	57.5 (40.5 to 65.3)	54.0 (38.5 to 62.5)	0.396
**Venous parameters:**	***n=*** **22**	***n=*** **11**	
Data quality, [subjective score 1.0 to 5.0]	4.5 (4.1 to 4.9)	4.0 (3.3 to 5.0)	0.435
Venous diameter, [MU]	55.5 (47.9 to 64.5)	61.9 (57.8 to 66.9)	0.285
Mean maximal venous dilation (mMD_ven_), [% baseline]	1.2 (0.7 to 1.8)	0.6 (0.2 to 0.9)	**0.075**
Time to maximal venous dilation (tMD_ven_), [s]	15.0 (10.6 to 20.6)	17.0 (7.5 to 18.3)	0.819
Venous dilation at flicker cessation (DFC_ven_), [% baseline]	0.6 (0.0 to 0.8)	0.0 (−0.7 to 0.1)	**0.100**
Venous reactive magnitude (RM_ven_), [% baseline]	2.1 (1.7 to 3.4)	1.8 (1.1 to 2.4)	0.236
Venous AUC during the flicker (AUC_ven_), [%*s]	8.7 (5.0 to 13.2)	0.5 (−3.9 to 5.8)	**0.047**
Mean maximal venous constriction (mMC_ven_), [% baseline]	−1.2 (−1.8 to −0.6)	−1.0 (−1.2 to −0.5)	0.479
Time to maximal venous constriction (tMC_ven_), [s]	57.5 (50.3 to 76.8)	50.0 (42.3 to 63.0)	0.268

*Bold values indicate p ≤ 0.1*.

**Figure 3:**
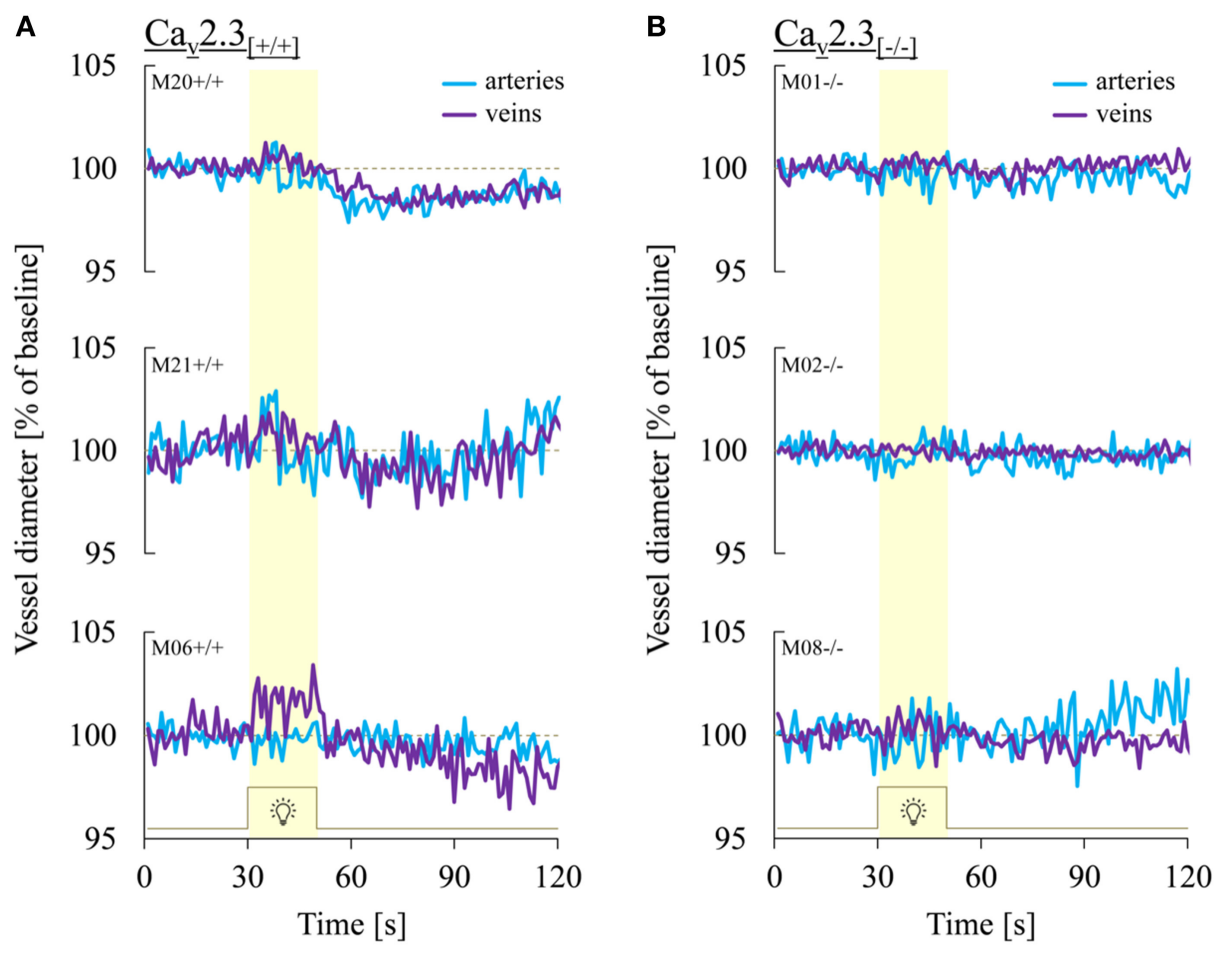
Representative retinal vessel responses recorded in Ca_v_2.3_[+/+]_ and Ca_v_2.3_[−/−]_ mice. Shown are typical patterns of arterial (turquoise) and venous (purple) responses to flicker light recorded in **(A)** Ca_v_2.3-competent (Ca_v_2.3_[+/+]_) and **(B)** Ca_v_2.3-deficient (Ca_v_2.3_[−/−]_) mice. Note that both arterial and venous retinal vessel responses in Ca_v_2.3_[−/−]_ mice were consistently reduced or completely absent.

**Figure 4:**
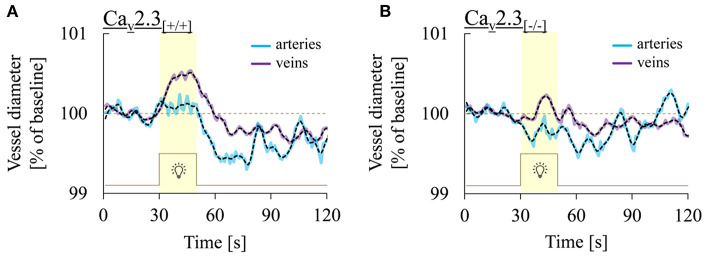
Comparison of retinal arterial and venous responses in the two genotypes. Shown are average arterial (turquoise) and venous (purple) responses to flicker light recorded in **(A)** Ca_v_2.3-competent [Ca_v_2.3_[+/+]_: *n* = 15 and 22 animals for arterial and venous responses, respectively] or **(B)** Ca_v_2.3-deficient [Ca_v_2.3_[−/−]_: *n* = 10 and 11 animals for arterial and venous responses, respectively]. Black dotted lines show the same data after smoothing with a 2-points FFT filter (cut-of frequency: 0.25) to remove high-frequency noise. Note that venous dilation during the flicker was consistently more pronounced than arterial dilation and that average responses in both vascular compartments were reduced in Ca_v_2.3_[−/−]_ mice.

Figure 3 shows three representative examples of retinal arterial and venous reactions to monochromatic flickering light recorded in Ca_v_2.3_[+/+]_ (Figure 3A) or Ca_v_2.3_[−/−]_ (Figure 3B) mice. Venous responses could be recorded in all 22 Ca_v_2.3_[+/+]_ and 11 Ca_v_2.3_[−/−]_ mice included in the present study, while recording of arterial responses was technically feasible in 15 of the Ca_v_2.3_[+/+]_ and 10 of the Ca_v_2.3_[−/−]_ mice. Based on the venous area under the curve (AUC_ven_), flicker stimulation evoked venous vasodilation (AUC_ven_>2.5%^*^s), venous vasoconstriction (AUC_ven_ < -2.5%^*^s) or no response (−2.5 < AUC_ven_ <2.5%^*^s) in 18 (82%), 3 (14%), or 1 (5%) of the Ca_v_2.3_[+/+]_ and 4 (36%), 4 (36%), or 3 (27%) of the Ca_v_2.3_[−/−]_ mice (*p* = 0.028, Table 2). In the arterial compartment, vasodilation, vasoconstriction or no response were observed in 14 (64%), 3 (14%) or 5 (23%) of the Ca_v_2.3_[+/+]_ and 4 (36%), 7 (64%), or 0 (0%) of the Ca_v_2.3_[−/−]_ mice (*p* = 0.008, Table 2). A comparison of average arterial and venous responses in the two genotypes is provided in Figure 4. Consistent with the results of our previous proof-of-principle study, retinal vessel responses in normal (Ca_v_2.3_[+/+]_) mice were smaller in magnitude than the corresponding responses observed in human subjects (17, 18, 26) but showed a similar biphasic shape with vasodilation during the flicker followed by vasoconstriction and gradual return to baseline or to a new steady-state after flicker cessation (Figure 4A).

On average, vessel dilation during the flicker was slower but more pronounced in the venous compartment, whereas vessel constriction after flicker cessation was stronger in the arterial compartment (Figure 4A). In addition, retinal vessel responses to flicker stimulation in both vascular compartments were altered in Ca_v_2.3-deficient mice (Figure 4B), as described in more detail in the following sections.

### Differences in Venous Responses to Flicker Stimulation

**Figure 5:**
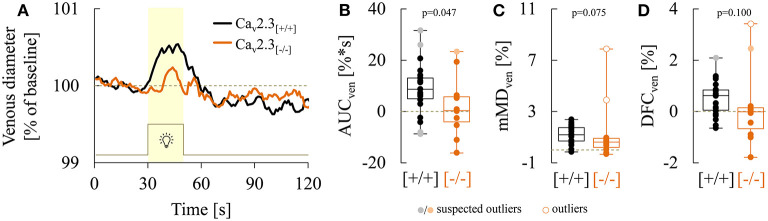
Comparison of venous responses recorded in Ca_v_2.3_[+/+]_ and Ca_v_2.3_[−/−]_ mice. **(A)** Average venous responses to flicker stimulation in Ca_v_2.3-competent (Ca_v_2.3_[+/+]_: black curve, *n* = 22 animals) and Ca_v_2.3-deficient (Ca_v_2.3_[−/−]_: orange curve, *n* = 11 animals) mice. **(B–D)** Boxplots comparing **(B)** venous area under the curve (AUC_ven_), **(C)** mean maximum venous dilation during the flicker (mMD_ven_), and **(D)** venous dilation at flicker cessation (DFC_ven_) in Ca_v_2.3_[+/+]_ (black) and Ca_v_2.3_[−/−]_ (orange) mice (same animals as in A). For remaining parameters see Table 3.

A comparison of average venous responses to flicker light measured in the two genotypes is provided in Figure 5A. Genetic ablation of Ca_v_2.3 channels was associated with a pronounced decrease of venous responses during the flicker (Figures 3B, 5A), as reflected in a significantly lower median venous AUC, which amounted to 0.5%^*^s (−3.9 to 5.8%^*^s) in Ca_v_2.3_[−/−]_ and 8.7%^*^s (5.0 to 13.2%^*^s) in Ca_v_2.3_[+/+]_ mice (*p* = 0.047, Figure 5B). There was also a tendency for maximum venous dilation in response to flicker to be reduced, with median values of 1.2% (0.7 to 1.8%) in Ca_v_2.3_[+/+]_ and 0.6% (0.2 to 0.9%) in Ca_v_2.3_[−/−]_ mice. However, because two of the Ca_v_2.3-deficient mice actually showed an increased venous dilation during the flicker when compared to Ca_v_2.3-competent mice (Figure 5C), the difference between both groups did not reach statistical significance (*p* = 0.075). Likewise, comparison of venous dilation at flicker cessation revealed no significant difference between the two genotypes (*p* = 0.100), even though relative vessel diameters were clearly reduced in all but two Ca_v_2.3_[−/−]_ mice (Figure 5D). On the other hand, there was little difference between the two genotypes with regard to the timing of venous dilation during the flicker (*p* = 0.819) or the magnitude (*p* = 0.479) and timing (*p* = 0.268) of venous constriction after flicker cessation (Figure 5A, Table 3).

### Differences in Arterial Responses to Flicker Stimulation

**Figure 6:**
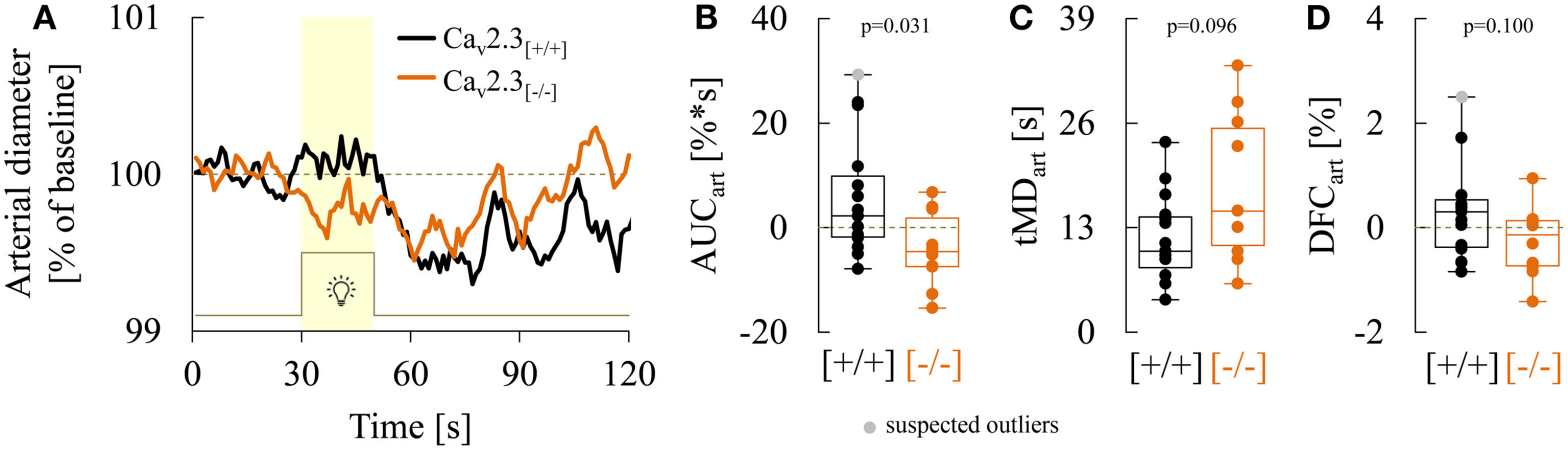
Comparison of arterial responses recorded in Ca_v_2.3_[+/+]_ and Ca_v_2.3_[−/−]_ mice. **(A)** Average arterial responses to flicker stimulation in Ca_v_2.3-competent [Ca_v_2.3_[+/+]_: black curve, *n* = 15 animals] and Ca_v_2.3-deficient [Ca_v_2.3_[−/−]_: orange curve, *n* = 10 animals] mice. **(B–D)** Boxplots comparing **(B)** arterial area under the curve (AUC_art_), **(C)** time of maximum arterial dilation during the flicker (tMD_art_) and, **(D)** arterial dilation at flicker cessation (DFC_ven_) in Ca_v_2.3_[+/+]_ (black) and Ca_v_2.3_[−/−]_ (orange) mice (same animals as in A). For remaining parameters see Table 3.

As illustrated in Figure 6A, genetic ablation of Ca_v_2.3 channels also resulted in alterations in retinal arterial responses, which consisted of a decrease and apparent initial reversal of vasodilation during the flicker. Quantitatively, this was reflected in a significant decrease of the arterial AUC during the flicker from a positive median value of 2.3% (−1.7 to 10.0%) in Ca_v_2.3_[+/+]_ to a negative median value of −4.5% (−7.3 to 1.9%) in Ca_v_2.3_[−/−]_ mice (*p* = 0.031, Figure 6B). The median time to maximal arterial dilation was also delayed from 10.0 s (8.0 to 14.3 s) in Ca_v_2.3_[+/+]_ to 15.0 s (10.8 to 25.3 s) in Ca_v_2.3_[−/−]_ mice, even though this difference did not reach statistical significance (*p* = 0.096, Figure 6C). In addition, arterial dilation at flicker cessation (Figure 6D) and the arterial reactive magnitude (Table 3) showed a tendency to be reduced in Ca_v_2.3-deficient compared to Ca_v_2.3-competent mice, although these differences remained below the threshold for statistical significance as well (*p* = 0.100 and *p* = 0.090, respectively).

## Discussion

Dynamic retinal vessel analysis is a powerful tool for non-invasive assessment of neurovascular coupling that remains to be widely adopted to experimental and preclinical murine animal models. Here, we used an adapted prototype of a non-contact retinal vessel analyzer to compare the responses to flicker stimulation in Ca_v_2.3-competent and -deficient mice.

### Retinal Vessel Analysis in Mice

Our findings confirm and extent the results of a previous proof-of-concept study (19) by showing that non-invasive assessment of murine retinal vessel responses is feasible and that it can be used to analyze changes in genetically modified mice. Even though the signal-to-noise ratio (SNR) of individual responses to flicker stimulation remains to be improved by further technical and methodological refinements (see below), the variability of the parameter values obtained in wildtype mice was not excessive and in line with the relatively high (inter- and intra-subject) heterogeneity of microvascular responses observed in other studies (29–31). Moreover, while the shape of the individual responses was often not well-resolved, especially the average results obtained are in good agreement with the expected distinct pattern of retinal vessel responses to flicker stimulation (32). For example, previous studies in rats and human subjects have shown that retinal arteries exhibit rapid vasodilation on flicker initiation followed by a maintenance phase with little change before rapidly dropping below the baseline after flicker cessation and slowly returning back to their original value (31–37). While the maintenance phase in our average arterial responses appeared to have been obscured by the lower SNR in mouse recordings, wildtype mice clearly exhibited rapid vasodilation at stimulation onset, rapid and pronounced vasoconstriction after flicker cessation and a subsequent slow return of the vessel diameter to baseline (Figure 6A). Venous responses in the retina are typically characterized by much slower vasodilation during the flicker stimulation followed by a slow return to baseline after flicker cessation (31–37), which is in line with the average venous response we observed in wildtype mice (Figure 5A, but see below). That said, like in our previous study (19), the responses to flicker light stimulation appeared to be more consistent and pronounced in veins as compared to arteries, which would not be expected based on the dominant working model that arteries are the main effector of NVC and is in actual contrast to the small and inconsistent venous dilation previously observed in the rat retina (35). A possible explanation with regard to the latter could be differences in the anesthetic regimen, as common doses of many anesthetics have been shown to alter NVC and block vasodilation of veins in response to neuronal activity [reviewed in Gao et al. (38)]. While the aforementioned studies in rats were performed under α-chloralose anesthesia (35), which has been shown to reduce the metabolic rate in cortical neurons by 50% or more (39, 40), we used ketamine/xylazine at 2/3 of the common intraperitoneal dose, which might have contributed to the more pronounced venous responses observed in the present study. Moreover, even though arterial and venous responses in the human retina have often been reported to be comparable in magnitude (34, 41–43), more recent work by us and others has consistently demonstrated that the magnitude of venous responses actually exceeds the magnitude of arterial responses, and that these difference further increase with increasing age (31, 36, 37). Indeed, from a fluid mechanical point of view and assuming the same volumetric flow in both arteries and veins but a higher velocity in the arterial compartment, veins should dilate more to accommodate the same blood volume. Other factors that need to be considered with regard to the lower SNR in murine recordings are that veins are usually larger than arteries and that they appear darker, which increases their contrast to the background, makes venous responses less susceptible to erroneous diameter estimations and facilitates their separation from background noise. We intent to use fluorescent dyes to further improve the threshold for detection and resolution of arterial responses in the murine retina in future studies.

Another interesting observation of the present study is the fact that venous vasodilation during the flicker was often followed by vasoconstriction after flicker cessation, which was less marked than arterial vasoconstriction but still clearly evident in the average responses (Figure 5A). While this may seem to be at odds with the absence of vasoconstriction after flicker cessation usually observed in human retinal veins (34, 41–43), a recent study on the smallest vessels in the human retina found instances of constriction in venules as well (29), suggesting that their responses may at least in part be actively generated by contractile mural cells present at the vessel wall. In support of this assumption, the tone of porcine retinal veins without identifiable smooth muscle cells has previously been shown to be modulated by vasoconstricting and vasodilating agents (44–46). Interestingly, basal tone and its modulation by vasoactive agents were abolished in the absence of extracellular Ca^2+^, while the L-type VGCC antagonist nifedipine reduced basal tone but had no effect on venous vasoreactivity, possibly pointing to a role of Ca^2+^-influx through non-L-type VGCCs (45). This makes it tempting to speculate that Ca_v_2.3 channels could be involved in the processes underlying venous vasoreactivity (but see next section), even though further studies will clearly be required to firmly establish whether the responses of veins in the mouse retina are attributable to passive stretch secondary to arterial vasodilation or at least in part actively generated by contractile cells like pericytes.

### Effects of Ca_v_2.3 Channel Ablation on Retinal Vessel Responses

Our results also suggest that Ca_v_2.3 channel dysfunction is associated with significantly reduced venous and possibly even inverted arterial responses to flicker stimulation. However, taking into account the limited SNR, further studies will be required to determine if the apparent inversion of arterial responses evident in Figures 6A,B was due to arterial vasoconstriction during the flicker or merely an artifact generated by background noise. That said, a reduction in the magnitude of vasodilation during flicker stimulation was clearly observed in both arteries and veins from Ca_v_2.3-deficient compared to wildtype mice, while the timing of vasodilation or the magnitude and timing of vasoconstriction after flicker cessation appeared to be much less affected. Attenuated vessel responses could reflect reduced light-induced neuronal activity with an associated decrease in metabolic demand or alterations in the coupling of neuronal activity to local vessel responses (i.e., impaired NVC). Our present data are clearly insufficient to draw firm conclusions with regard to the underlying mechanisms. However, previous electroretinographic (ERG) recordings from the isolated murine and bovine retina indicate that genetic or pharmacological ablation of Ca_v_2.3 channels produces no drastic neuronal dysfunction and actually increases the ERG b-wave, presumably due to reduced GABAergic feedback inhibition of rod bipolar cells (47–51). Also, while the decrease in retinal vessel dilation during flicker stimulation could be accounted for by reduced neuronal activity, the partial reversal of arterial responses in Ca_v_2.3-deficient mice is more difficult to explain in terms of altered neuronal activity alone. As such, we propose that the observed reduction of retinal vessel responses reflects—at least in part—impaired NVC due to changes in one or more of the signaling pathways within the neurovascular unit. On first sight, this may appear difficult to reconcile with previous findings that Ca^2+^ influx through Ca_v_2.3 channels in cerebral vessels actually contributes to vasoconstriction after experimental subarachnoid hemorrhage (SAH) (20). However, it has also been shown that Ca_v_2.3 channels are not normally expressed in these vessels and that subarachnoid blood degradation products like hemoglobin may be required to induce their functional upregulation (12, 20, 52). As such, our findings could point to a role of Ca_v_2.3 channels in neurons, astrocytes, capillary pericytes and/or endothelial cells for NVC. For example, Ca_v_2.3 channels have been shown to be expressed in cultured astrocytes (53) and astrocyte Ca^2+^ signaling has implicated in the modulation of basal tone and pressure-induced vascular responses of retinal arteries and veins (54). The inversion of NVC after SAH has been linked to changes in astrocyte Ca^2+^ signaling (55) as well, and sustained exposure to pathophysiologically relevant concentrations of the hemoglobin degradation product bilirubin has been shown to impair Ca_v_2.3 channel function (56), which could conceivably promote changes in NVC similar to those observed in the present study. This could have important clinical implications with regard to the proposed use of Ca_v_2.3 channel antagonists for the treatment of cerebral vasospasm (20), as suppression of these channels in non-vascular cell types of the NVU could possibly further impair NVC. In any case, further studies on the role of Ca_v_2.3 channels in different cell types belonging to the NVU will clearly be required to delineate their contribution to NVC under physiological and pathophysiological conditions. In particular, combination of RVA with techniques for assessment of inner retinal signaling could be used to confirm or refute our hypothesis that altered retinal vessel responses in Ca_v_2.3-deficient mice cannot be accounted for by changes in neuronal activity alone. More broadly speaking, non-invasive assessment of murine retinal vessel responses by RVA represents a powerful tool for preclinical research on NVC, which could not only provide insight into the therapeutic action of currently used drugs like nimodipine (13) but also help to identify novel targets for improved treatment strategies. However, a potential limitation of RVA in its current form that should be mentioned is that evaluation of retinal vessel diameters and responses without a mydriaticum is not technically feasible, so that a (differential) effect of tropicamide application on Ca_v_2.3-competent and –deficient mice cannot be excluded. Although previous studies indicate that pharmacologic mydriasis with tropicamide has no effect on retinal microcirculation in the macula and peripapillary region (57), we are currently working on a refined prototype with infrared cameras to overcome the need for mydriasis and further evaluate its impact on retinal vessel parameters.

## Conclusion

To our knowledge, this is the first study using a novel, non-contact analysis technique to investigate retinal vessel responses in VGCC-deficient mice. The observed changes in mice lacking Ca_v_2.3 VGCCs raise the possibility that these channels are involved in NVC and deserve further investigation.

## Data Availability Statement

The original contributions presented in the study are included in the article/supplementary material, further inquiries can be directed to the corresponding author/s.

## Ethics Statement

The animal study was reviewed and approved by the institutional and governmental committees on animal care (Landesamt für Natur, Umwelt und Verbraucherschutz [LANUV] Nordrhein—Westfalen, Recklinghausen, Germany; 84-02.04.2016.A4555) approved all experiments described in the text, which were conducted in accordance with accepted standards of humane animal care, as reported in the UFAW handbook on the care and management of laboratory animals.

## Author's Note

Part of this study was presented at the 72nd Annual Meeting of the German Society of Neurosurgery (DGNC), Joint Meeting with the Japan Neurosurgical Society (JNS), June 2020, Germany.

## Author Contributions

FN, TS, and WA conceived, designed, and performed the experiments. FN and WA first drafting of the manuscript and illustrations. FN, JL, WA, KK, and OS data acquisition. FN, KK, RHLH, YT, UL, GS, TS, WA, and OS analysis and interpretation of data. KK, RHLH, YT, UL, HC, JH, GS, and TS critical review of the manuscript. The final manuscript was critically revised and approved by all authors.

## Conflict of Interest

The authors declare that the research was conducted in the absence of any commercial or financial relationships that could be construed as a potential conflict of interest.

## Abbreviations

AUC: area under the curve
AUC_art_: arterial area under the curve during the flicker
AUC_ven_: venous area under the curve during the flicker
DFC_art_: arterial dilation at flicker cessation
DFC_ven_: venous dilation at flicker cessation
DVA: dynamic vessel analyzer
ERG: electroretinographic
mMC_art_: mean maximal arterial constriction after flicker cessation
mMC_ven_: mean maximal venous constriction after flicker cessation
mMD_art_: mean maximal arterial dilation in response to the flicker
mMD_ven_: mean maximal venous dilation in response to the flicker
MU: arbitrary measuring units
NVC: neurovascular coupling
NVU: neurovascular unit
ONH: optic nerve head
RM_art_: arterial reactive magnitude
RM_ven_: venous reactive magnitude
RVA: retinal vessel analysis
SAH: subarachnoid hemorrhage
tMC_art_: time to maximal arterial constriction after flicker cessation
tMC_ven_: time to maximal venous constriction after flicker cessation
tMD_art_: time to maximal arterial dilation during the flicker
tMD_ven_: time to maximal venous dilation during the flicker
VGCCs: voltage-gated calcium channels.
